# Ventral tegmental area dopaminergic action in music therapy for post-traumatic stress disorder: A literature review

**DOI:** 10.3389/fpsyg.2022.1014202

**Published:** 2022-10-10

**Authors:** Meng Ning, Shizhe Wen, Peiling Zhou, Changzheng Zhang

**Affiliations:** ^1^School of Music, Huainan Normal University, Huainan, China; ^2^School of Educational Sciences, Lingnan Normal University, Zhanjiang, China

**Keywords:** music therapy, post-traumatic stress disorder, symptom improvement, ventral tegmental area, dopaminergic action

## Abstract

Post-traumatic stress disorder (PTSD) is a debilitating sequela of extraordinary traumatic sufferings that threaten personal health and dramatically attenuate the patient's quality of life. Accumulating lines of evidence suggest that functional disorders in the ventral tegmental area (VTA) dopaminergic system contribute substantially to PTSD symptomatology. Notably, music therapy has been shown to greatly ameliorate PTSD symptoms. In this literature review, we focused on whether music improved PTSD symptoms, based on VTA dopaminergic action, including the effects of music on dopamine (DA)-related gene expression, the promotion of DA release and metabolism, and the activation of VTA functional activities. In addition, the strengths and limitations of the studies concerning the results of music therapy on PTSD are discussed. Collectively, music therapy is an effective approach for PTSD intervention, in which the VTA dopaminergic system may hold an important position.

## Introduction

Post-traumatic stress disorder (PTSD) is a debilitating psychiatric disorder that occurs following exposure to extraordinary actual or threatened trauma (such as death, serious injury, or sexual violation), with four cardinal symptomatic clusters according to criteria of the Diagnostic and Statistical Manual of Mental Disorders (DSM)-5: re-experiencing (e.g., intrusive trauma-related imagery and reliving in nightmares), avoidance (e.g., avoiding reminders of the traumatic event), negative cognitions and moods (e.g., depression and anxiety), and arousal (e.g., prolonged hypervigilance that causes irritability or frequent outbursts of anger, difficulty in concentrating, trouble falling asleep and exaggerated startle response); and the duration of the disturbance lasts more than 1 month (American Psychiatric Association, 2013; Bisson et al., 2015; Shalev et al., 2017). PTSD remarkably impairs personal and social functions, causing, for example, obstacles in interpersonal relationships (Bisson et al., 2015; Yehuda et al., 2015; Shalev et al., 2017), family instability (Jordan et al., 1992), substance use disorders (Bisson et al., 2015; Hakvoort et al., 2020), high risks of suicidal ideation (Bisson et al., 2015; Brown et al., 2020), and considerable cost-effectiveness of clinical treatments (Bisson et al., 2015; Von Der Warth et al., 2020). In addition, the biological vulnerability to PTSD may be transmitted across generations through epigenetic processes (Ramo-Fernández et al., 2015; Yehuda et al., 2015).

It is estimated that the overall lifetime prevalence of PTSD ranges from 1.3 to 12.2% in civilians (Zhou et al., 2021), with higher prevalence in some populations such as in soldiers and rape survivors (Bisson et al., 2015; Shalev et al., 2017; Baranyi et al., 2018; Eiset et al., 2021). The reason why an individual is susceptible to PTSD while others exhibit resilience or recovery remains largely unknown.

There are several evidence-based therapies recommended for PTSD, such as psychological, pharmacological, and physiotherapeutic interventions (Bisson et al., 2015; Yehuda et al., 2015). Although such therapies are empirically validated, their actual feasibility remains highly debated (Harvey et al., 2003; Bradley et al., 2005; Wilson et al., 2018). None of the available methods are uniformly successful, and all of the methods have been reported with side effects (Masand and Gupta, 2002; Guina et al., 2015; Akiki and Abdallah, 2018). Moreover, some therapies are contraindicated in children, pregnant women, and individuals with special requirements, such as due to liver/kidney dysfunctions (Blanaru et al., 2012).

Notably, music therapy has been shown to exhibit significant effects on trauma symptoms (e.g., it improves wellbeing and sleep quality) in traumatized refugees (Jespersen and Vuust, 2012; Beck et al., 2018a, 2021). There is abundant theoretical and empirical evidence concerning the beneficial effects of music therapy on PTSD patients (**Table 3**). Considering that PTSD is strongly associated with mesolimbic dopaminergic dysfunction (Zhou et al., 2021), the aim of the present review was to detail whether and how music therapy affects PTSD associated with VTA dopaminergic action.

## Method of literature search

**Figure 1:**
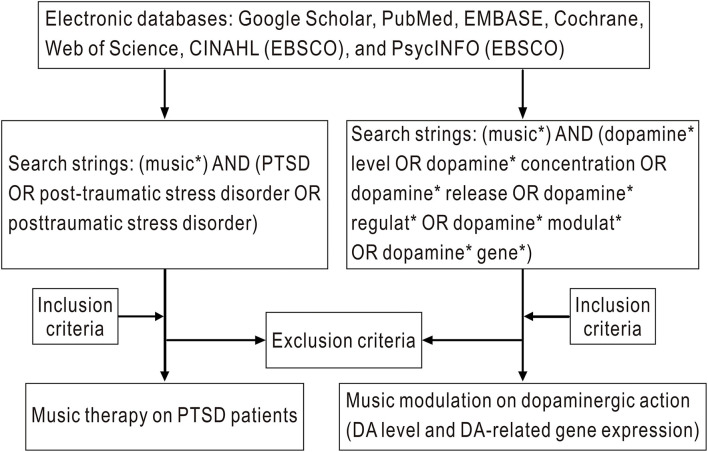
Flow diagram detailing the primary steps of the literature search.

The previous studies published between January 1, 1960 and July 20, 2022 were searched using electronic databases, such as Google Scholar, PubMed, EMBASE, Cochrane, Web of Science, CINAHL (EBSCO), and PsycINFO (EBSCO). For studies involving music therapy on PTSD, the search strings were (music^*^) AND (PTSD OR post-traumatic stress disorder OR posttraumatic stress disorder). The inclusion criteria were as follows: (1) human studies, (2) a clear diagnosis of PTSD, and (3) music therapy used to treat the PTSD symptoms. To identify studies of music modulation on dopaminergic action, the search strings were (music^*^) AND (dopamine^*^ level OR dopamine^*^ concentration OR dopamine^*^ release OR dopamine^*^ regulat^*^ OR dopamine^*^ modulat^*^ OR dopamine^*^ gene^*^). The inclusion criteria were as follows: (1) either human studies or those in rats or mice, (2) studies that evaluated DA levels, DA metabolite levels, or DA-related gene expression following music therapy, and (3) with a clear method for measuring DA level or DA-related gene expression. The exclusion criteria were as follows: (1) review/meta-analyses, (2) non-full-text publications, or (3) articles not written in English. The flow diagram for the literature search is shown in Figure 1.

The title and abstract of each study were first screened to identify potential literatures on this topic. Full texts were then assessed for eligibility based on the inclusion and exclusion criteria. The articles selected to evaluate the effects of music therapy on PTSD and music modulation on dopaminergic action (DA level and DA-related gene expression) are presented in Tables 1–**3**.

**Table 1:** Dopamine (DA)-related candidate genes associated with listening to music.

**Gene**	**Common name**	**Association with music**	**Association with DA**
*DRD2* (or *D2R, D2DR*)	DA receptor D2	Associated with mood improvements after musical stimulus (Quarto et al., 2017); inconclusive associations with musical traits (Ukkola et al., 2009; Mariath et al., 2017).	Associates with dopaminergic activities.
DRD4 (or *D4R, D4DR*)	DA receptor D4	Increased expression in musicians (Emanuele et al., 2010); no association with musicality (Mariath et al., 2017).	Associates with dopaminergic activities.
*COMT*	Catechol-O-methyltransferase	Associated with improvising, and weakly associated with pitch recognition (Ukkola et al., 2009); inconclusive associations with musical aptitudes (Mariath et al., 2017).	Inactivates DA, and is involved in the regulation of extra-synaptic DA levels.
*SNCA*	alpha-synuclein gene	Upregulated after listening to music, which is associated with musical aptitude (Kanduri et al., 2015b).	Involved in DA metabolism and synaptic plasticity (Järvelä, 2018).
*GATA2*	GATA binding protein 2	Associated with musical aptitude (Oikkonen et al., 2015).	Regulates SNCA in dopaminergic neurons (Järvelä, 2018).
*RTN4*	Reticulon 4	Upregulated after listening to music (Kanduri et al., 2015b).	Involved in DA secretion, transport, and signal transduction (Kanduri et al., 2015b).
*SLC6A8*	Solute Carrier Family 6 Member 8	Upregulated after listening to music (Kanduri et al., 2015b).	Primarily involved in DA secretion, transport, and signaling (Kanduri et al., 2015b).
*PPP2R3A*	Protein phosphatase 2 regulatory subunit Bα	Downregulated after listening to music, but upregulated after musical performance (Kanduri et al., 2015a,b).	Highly expressed in the striatum, which is involved in DA metabolism (Järvelä, 2018).
*RGS2*	Regulator of G protein signaling gene family-2	Upregulated after listening to music (Kanduri et al., 2015a,b).	Modulates DA signaling and regulates intracellular signaling of G protein-coupled receptors (Järvelä, 2018).
*RGS9*	Regulator of G protein signaling gene family-9	Involved in song perception and production processes; associated with musical aptitude (Liu et al., 2016).	Modulates DA signaling and regulates intracellular signaling of G protein-coupled receptors (Rahman et al., 2003; Järvelä, 2018).

## Results

### Music alters dopaminergic gene expression

Music listening and performance have been reported to alter the expression of multiple DA-related genes (Table 1). A recent study found that hsa-miR-23a was upregulated in music listeners (Nair et al., 2021), and as hsa-miR-23a was associated with dopaminergic neuronal activation and striatal DA stabilization (Nair et al., 2003, 2021), music may strengthen dopaminergic actions and induce feelings of pleasure (Menon and Levitin, 2005; Stegemöller, 2014; Zatorre, 2015).

The alpha-synuclein gene (*SNCA*), which is located in the region with the strongest linkage to musical aptitude on chromosome 4q22.1 (Kanduri et al., 2015b), is significantly up-regulated in individuals listening to and/or performing music (Järvelä, 2018; Nair et al., 2021). As *SNCA* is closely linked to DA metabolism, music therapy may regulate the dopaminergic pathway (Järvelä, 2018). *GATA2* (encoding GATA binding protein 2) is abundantly expressed in dopaminergic neurons and is also up-regulated following stimuli with music (Järvelä, 2018). Interestingly, this gene regulates *SNCA* expression (Kanduri et al., 2015b), indicating that there may be a synergistic effect in both genes due to musical stimuli. It should be noted that over-expression of *SNCA* and *GATA2* has been implicated in Parkinson's disease (Somayaji et al., 2021) and hematopoietic diseases (Zhou et al., 2019), respectively. We hypothesize that music-mediated up-regulation of these genes is limited to certain brain regions (e.g., music-mediated up-regulation principally in VTA) or the overall expression level is far below pathopoiesia.

Several other genes involved in DA secretion, transport, and signaling are also up-regulated following listening to music and/or musical performance. For example, *RTN4* is involved in DA secretion (Kanduri et al., 2015b); *SLC6A8* is associated with DA regulation, secretion, and transport (Kanduri et al., 2015b); *PPP2R3A* is involved in DA metabolism (Järvelä, 2018); and *RGS9* is involved in the regulation of intracellular signaling in dopaminergic neurons (Rahman et al., 2003; Järvelä, 2018). However, some researchers reported inconsistent changes in the expressions of DA-related genes. For example, *DRD2* and *DRD4* have been found to have associations (Ukkola et al., 2009; Quarto et al., 2017) and inconclusive relationships with musical aptitudes (Ukkola et al., 2009; Mariath et al., 2017).

Collectively, several DA-related genes are up-regulated upon listening to music, and may facilitate dopaminergic release, activate the reward pathways, and ultimately correlate with pleasant feelings (Stegemöller, 2014; Zatorre, 2015; Moraes et al., 2018).

### Music affects DA release and metabolism

Endogenous DA release was markedly increased when individuals listened to pleasurable music; however, the DA release pattern was significantly different in different anatomical pathways, e.g., music-induced DA release in the caudate was more involved during the anticipation of listening to music, whereas DA release in the nucleus accumbens (NAc) was more involved during listening to music (Salimpoor et al., 2011).

Studies from animals and human beings have indicated that music listening enhanced the DA levels in the brain, such as increases in DA in the NAc, VTA, prefrontal cortex (PFC), and neostriatum (Sutoo and Akiyama, 2004; Menon and Levitin, 2005; Feduccia and Duvauchelle, 2008; Tasset et al., 2012). However, other studies reported no differences in DA levels in the NAc, piriform cortex, motor cortex, insular cortex, and somatosensory cortex after musical stimuli (Sutoo and Akiyama, 2004). Interestingly, dual pharmacological manipulation of DA through the use of a DA precursor (levodopa) and a DA antagonist (risperidone) positively and negatively affects human responses to music, thus, indicating a causal role of DA function in musical pleasure (Ferreri et al., 2019). Examples of DA and dopaminergic metabolite changes in response to musical stimuli are listed in Table 2.

**Table 2:** Alterations of DA and its metabolites after music treatment.

**References**	**Subjects**	**Methods**	**Results**
Yamamoto et al. (2003)	Six male participants aged 24.0 ± 4.1 years	Participants listened to the music (with slow and fast rhythm). The plasma DA was measured by high performance liquid chromatography (HPLC).	Music induced no significant alterations in the plasma DA level.
Salimpoor et al. (2011)	Eight participants of both males and females, between 19 and 24 years of age	Participants listened to pleasurable music. DA release was examined by positron emission tomography (PET) and functional magnetic resonance imaging (fMRI).	Endogenous DA was released in the striatum at peak emotional arousal during music listening. DA release in the caudate was associated with music anticipation, while DA release in the NAc was associated with music experience.
Sutoo and Akiyama (2004)	Spontaneously hypertensive male rats at 12 weeks of age	Rats were exposed to Mozart's music (K.205) at loudness of ~ 65 dB for 120 min. After the musical stimuli, the DA levels in brain slices were examined by quantitative immunohistochemistry.	Music increased DA levels in the neostriatum, whereas the DA levels in other regions (such as NAc, piriform cortex, motor cortex, insular cortex and somatosensory cortex) were not significantly altered.
Feduccia and Duvauchelle (2008)	Male Sprague-Dawley rats, with 200–250 g body weight	Rats were treated with MDMA in the conditioning chambers accompanied with music (the Very Best Euphoric House Breakdown) at 65–75 dB for 40 min on days 1, 3, 5 vs. saline treatment without music on days 2, 4, 6. The DA level was examined using *in vivo* microdialysis with HPLC.	Music significantly promoted DA release in the NAc of MDMA-treated rats.
Polston et al. (2011)	Female Sprague-Dawley rats, with 225–275 g body weight	Rats were administered methamphetamine in the conditioning chambers, accompanied with music (Miles Davis's “Four”) at 65–75 dB for 90 min per secession for 7 days. DA and its metabolites (dihydroxyphenylacetic acid, DOPAC or homovanillic acid, HVA) were measured by *in vivo* microdialysis with HPLC before and after methamphetamine re-treatment in the presence of music.	Musical cues significantly increased DA levels in the basolateral amygdala and the NAc in methamphetamine-dependent rats, with no differences in the levels of DOPAC or HVA in the basolateral amygdala or NAc.
Tasset et al. (2012)	Male 2-month-old Wistar rats, with 200–250 g body weight	Mozart's music (K.488) was played 4 h daily for 4 days, with an average sound level of 65 dB. DA levels in the brain tissues were measured by enzyme-linked immunosorbent assay (ELISA).	Music significantly increased DA levels in the PFC, striatal nucleus, and mesencephalon.
Moraes et al. (2018)	Male Wistar rats, with 307.1 ± 12.4 g body weight and 15.0 ± 0.5 weeks old	Rats were subjected to Mozart's music (K.488) for 4 h daily for 4 days, with a sound level of 65–75 dB. Levels of DA and its metabolite, DOPAC, were measured by HPLC.	Music significantly increased DA levels in both the caudate putamen and the NAc, while DOPAC was markedly increased in the NAc only.
Luo et al. (2021)	Male Sprague-Dawley rats, with 250–300 g body weight.	Rats (in diabetic retinopathy with depression model) were subjected to Mozart's music (K.488) for 1 h daily for 8 weeks. Hippocampal DA level was measured by ELISA.	Music significantly promoted candesartan (a kind of drug for preventing retinopathy)-evoked DA release in hippocampus.

The DA from animal samples was measured mainly by HPLC (Feduccia and Duvauchelle, 2008; Polston et al., 2011; Moraes et al., 2018), ELISA (Tasset et al., 2012), and quantitative immunohistochemistry (Sutoo and Akiyama, 2004), while two studies performed in human samples used HPLC (Yamamoto et al., 2003), and PET and fMRI (Salimpoor et al., 2011).

### Music activates VTA function

Music-induced VTA activation has been detected with fMRI (Menon and Levitin, 2005). Music also activated the connectivity between the VTA and other regions, such as the NAc, hypothalamus, insula, orbitofrontal cortex, and bilateral inferior frontal cortex (Menon and Levitin, 2005). However, no data are available to illustrate whether and how music affects VTA neuronal activities, such as dopaminergic neuron firing or burst characteristics [burst represents a periodic high-discharge firing that produces robust dopamine release (Zhou et al., 2021)], or electrophysiological properties between dopaminergic and other neurons, such as long-term potentiation [persistent synaptic strengthening after stimulation (Ungless et al., 2001)].

### Summary of music therapy on PTSD

Music therapy has been reported to have ameliorative effects on almost all PTSD symptomatic clusters. Examples of music therapy for PTSD patients are listed in Table 3.

**Table 3:** Examples of music therapy for the patients of post-traumatic stress disorder (PTSD).

**References**	**Participant country(-ies)**	**PTSD individuals**	**Research design**	**Mode of music therapy**	**Music induction**	**Therapeutic effects**
Bensimon et al. (2008)	Israel	Six male PTSD soldiers aged 20–23 years, with unknown PTSD diagnosis criteria	Mixed design Two variables with multiple measurements	GMT. The participants and the therapists sat and played any musical instrument (drums and other instruments); 90-min session; weekly for 16 weeks.	Active performance, live music	Reduction in PTSD symptoms; increase in interpersonal intimacy and sense of self-control.
Precin (2011)	USA	A 20-year-old female with PTSD (no indicated diagnosis criteria)	Simple design A case study	The participant performed heavy metal music, which was full of high-powered driving rhythms, and she also created songs with the therapist to express her feelings.	Active performance, live music	Improvements in PTSD symptoms and rehabilitative power to work.
Bensimon et al. (2012)	Israel	Six male PTSD soldiers aged 20–23 years, with unknown PTSD diagnosis criteria	Mixed design Two variables with multiple measurements	GMT. The participants and the therapists sat and played any musical instrument (drums and other instruments); 90-min session; weekly for 16 weeks.	Active performance, live music	Decrease in the reflections of traumatic emotions, and increase in expressions of nontraumatic feelings.
Blanaru et al. (2012)	Israel	Thirteen individuals with average age of 45.7 years (eight males and five females), with PTSD diagnosed by DSM-IV	Mixed design Two variables with pre-post tests	The music was a slow melody with minor harmony, which was played for ~40 min each night for 1 week.	Receptive listening, prerecorded music	Amelioration of the severity of depression, anxiety and sleep disorder.
Carr et al. (2012)	UK	Sixteen participants aged 20–57 years (seven males and nine females), with PTSD diagnosed by the Clinician-Administered PTSD Scale	Mixed design Two groups with pre-post tests	GMT. The participants had free access to various musical instruments and were encouraged to improvise with the therapists; 1-h session weekly for 10 weeks.	Active performance, live music	Improvement in the PTSD symptoms, such as PTSD severity and depression.
Maack (2012)	German, USA, Swedish, Swiss, Turkish, Spanish, Croatian, Danish.	One hundred and thirty-six female participants aged 18–64 years, with PTSD diagnosed by the Disorder of Extreme Stress	Mixed design Four groups with pre-post tests	GIM with individualized music. The therapy was guided by the therapist, and the intervention time varied among participants.	Receptive listening, prerecorded music	Improvement in PTSD symptoms and alleviation in interpersonal problems, with follow-up effects at 1 year after the intervention.
Lightstone et al. (2015)	Canada	A male veteran, over 50 years of age, with PTSD diagnosed decades ago (no indicated diagnosis criteria)	Simple design A case study	Remotely-delivered music therapy. The interventions included music improvisation and instrumental playing, guided by the therapist.	Active performance, live music	Improvement in PTSD symptoms, life quality, emotional stabilization, and enabling the participant to accept psychotherapy (such as EMDR).
Zergani and Naderi (2016)	Iran	Forty veterans of unknown age and sex. The PTSD criteria were based on files from the hospital records.	Randomized control trial Two groups with pre-post tests	The participants were treated with traditional Iranian musical instruments. This therapy contained ~20 sessions of 45 min each for 45 days.	Receptive listening, prerecorded music	Improvement in the quality of life and reduction in anxiety, with follow-up effects at 1 month after the intervention.
Story and Beck (2017)	USA	Five female veterans aged 28–69 years, with PTSD diagnosed by the PTSD self-reported check list (PCL-5)	Mixed design Two variables with pre-post tests	GIM with individualized music. The participants were treated during 90-min weekly sessions for 10 weeks.	Receptive listening, prerecorded music	Alleviations of the PTSD symptoms.
Rudstam et al. (2017)	Sweden and other countries	Ten females aged 28–54 years, with PTSD diagnosed by PCL-5	Mixed design Two groups with pre-post tests	Trauma-focused group music and imagery (TFGrpMI) with individualized music. The treatment was administered in groups; 12 sessions of 2.5 h weekly.	Receptive listening, prerecorded music	Decrease in PTSD symptoms and increase in life quality, with follow-up effects at 3 months after the intervention.
Pezzin et al. (2018)	USA	Forty veterans aged 22–76 years (thirty-six males and four females) with PTSD diagnosed using the PTSD Checklist Civilian (PCLC)	Randomized control trial. Two groups with pre-post tests	Music-instruction intervention. The participants received music instruction and played guitar; 1-h weekly sessions for 6 weeks.	Receptive listening, prerecorded music	Reduction in PTSD symptom severity and depressive phenotypes.
Beck et al. (2018a)	Syria, Afghanistan, Iraq, Iran etc.	Seventy refugees aged 18–67 years (with unknown sex) with PTSD diagnosed by ICD-10, DSM-V, or DSM-IV-TR	Randomized control trial. Two groups with pre-post tests	Trauma-focused music and imagery with individualized music. The therapy included 60-min weekly sessions for 4–6 months.	Receptive listening, prerecorded music	Alleviation of PTSD symptoms.
Beck et al. (2018b)	Iraq, Afghanistan, Syria, Iran	Sixteen participants aged 19–60 years (ten males and six females), with PTSD diagnosed by ICD-10, DSM-V, or DSM-IV-TR	Simple design A non-controlled pre-post test study	GIM with individualized music. The treatment was provided in 1-h sessions for 15–48 weeks.	Receptive listening, prerecorded music	Improvement in the trauma symptoms, sleep quality, life quality, and social function.
Macfarlane et al. (2019)	Netherland	Thirteen male prisoners aged 25–54 years, with PTSD diagnosed by DSM-IV-TR	Simple design A non-controlled pre-post test study	Short-term Music therapy Attention and Arousal Regulation Treatment (SMAART). The participants performed musical (rhythmic and breathing) assignments; six individual sessions of a maximum of 60 min.	Active performance, live music	Decrease in arousal and improvement in attention. Some participants did not meet the threshold for a PTSD diagnosis.
Hirschberg et al. (2020)	USA	Ten veterans aged 28–52 years (nine males and one female), with PTSD diagnosed by Intensive Clinical Program (ICP)	Simple design A non-controlled pre-post test	Collaborative songwriting intervention. The participants co-wrote songs and listened to the songs; 75-min sessions daily for 5 weeks.	Active performance, live music	Improvement in PTSD symptoms, particularly in numbing and hyperarousal phenotypes.
Hakvoort et al. (2020)	Netherlands	Twelve participants aged 44 ± 12.9 years (six males and six females), with PTSD diagnosed by DSM-5. Six of the participants dropped out of the study (three males and three females remained).	Simple design A non-controlled pre-post test	SMAART. The participants received weekly 1-h music sessions for 6 weeks.	Active performance, live music	Improvements in PTSD symptom severity, such as hyperarousal, negative moods and cognition, and attention problem.
Beck et al. (2021)	Syria, Iraq, Bosnia, Kosovo, Lebanon, Iran, Afghanistan, Somalia, Sri Lanka, or Chechnya.	Fifty-eight participants aged 18–68 years (thirty-four males and twenty-four females), with PTSD diagnosed by ICD-10	Randomized control trial Two groups with pre-post tests	Trauma-focused GIM with individualized music. The intervention included a 1-h weekly session for 16 weeks.	Receptive listening, prerecorded music	Decrease in trauma symptoms, enhancement in wellbeing, and reduction of psychoform dissociation, with follow-up effects at 6 months after the intervention.
Pourmovahed et al. (2021)	Iran	Forty-five mothers of premature neonates aged an average of 28 years, with PTSD diagnosed by the Prenatal PTSD Questionnaire	Randomized control trial Two groups with pre-post tests	The non-verbal music included the sound of rain, sea, and nature with a slow, gentle and soothing rhythm. The participants listened to the music for 20–30 min daily for 2 weeks.	Receptive listening, prerecorded music	Decrease in PTSD symptom severity.
Rudstam et al. (2022)	Sweden and other countries	Forty-five female participants with average ages of 43.7 ± 9.93, with PTSD diagnosed by PCL-5. Five participants dropped out during the intervention.	Randomized control trial Two groups with pre-post tests	TFGrpMI with classical music from western traditions. The therapy consisted of weekly 2.5-h sessions for 12 weeks.	Receptive listening, prerecorded music	Decrease in PTSD symptom severity, with follow-up effects at 3 months after the intervention.

Although the results reported the positive effects of music therapy on PTSD, there are several drawbacks in the studies, such as their small sample sizes, lack of rigorous controls, and the lack of details of the music therapy.

Several studies reported that PTSD patients listening to music experienced remarkable reductions in PTSD symptoms (Blanaru et al., 2012; Pourmovahed et al., 2021), even when experienced through remotely delivered modalities (Lightstone et al., 2015). In general, group music therapy (GMT) may be more effective than single music treatment for the reduction of the severity of PTSD symptoms and improvement in quality of life (Bensimon et al., 2012; Carr et al., 2012; Macfarlane et al., 2019; de Witte et al., 2022).

Different modalities of music therapies exert different effects on PTSD phenotypes aside from the cardinal symptoms. Guided imagery and music (GIM) significantly decreases the symptoms of dissociation (Maack, 2012; Story and Beck, 2017); GIM in the trauma-focused group of patients enhanced PTSD patients' quality of life and wellbeing (Rudstam et al., 2017; Beck et al., 2021); songwriting treatment reduced the numbing and depressive symptoms of PTSD patients (Coulter, 2000; Precin, 2011; Hirschberg et al., 2020), and playing musical instruments, such as drums or guitars, increased the sense of openness and togetherness, and facilitated non-intimidating access to traumatic memories in PTSD patients (Bensimon et al., 2008; Pezzin et al., 2018).

Collectively, music therapy has idiosyncratic merit and benefits for PTSD intervention. Moreover, the therapeutic modality may also affect the effect of treatment.

### Music ameliorates PTSD cardinal symptoms

#### Intrusion

Intrusion represents unwanted distressing memories or cues that evoke “flashbacks” of the traumatic events, causing the patient to not be able to distinguish the past from the present (Bisson et al., 2015; Shalev et al., 2017; Fenster et al., 2018). Carr et al. (2012) found that music therapy eliminated dissociative flashbacks in PTSD patients. Music may establish “a safe space” in the brain that strengthens the tolerance for traumatic experiences (Bensimon et al., 2008), helps PTSD patients remain grounded, and helps them distinguish the present moment from the past traumatic events (Volkman, 1993; Orth, 2001; Bensimon et al., 2008).

#### Avoidance

Avoidance includes the intentional forgetting of past events, or rejecting to face the cues that might associate with traumatic events (Bisson et al., 2015; Shalev et al., 2017; Fenster et al., 2018). Music therapy is an effective approach to addressing the avoidant behavior of PTSD sufferers. Music improvization requires active participation, not just passive acceptance (Volkman, 1993; Orth, 2005), which requires the participants to engage and foster group commitments, and avoid distressing memories or cues associated with their trauma (Orth, 2005; Bensimon et al., 2008; Carr et al., 2012). Remarkably, during group music actions, PTSD patients listen to his/her playing, focus on another's creation, and enjoy the entire atmosphere simultaneously, which may distract from one's own traumatic memories (Carr et al., 2012; de Witte et al., 2022).

#### Negative recognition and mood

Negative cognition and mood include the inability to experience positive emotion and persistent negative beliefs (Bisson et al., 2015; Shalev et al., 2017; Fenster et al., 2018). Music is a helpful way of emotional expression, particularly on the distress caused by trauma. For example, playing musical instruments or singing songs elicit strong emotional responses to control negative moods, relieve bad feelings, and release physical energy (Orth, 2005; Bensimon et al., 2008; Carr et al., 2012). Music has been widely used to express feelings of anger and irritability by encouraging the tolerance of silence and loud sounds (Volkman, 1993; Orth, 2005; Bensimon et al., 2008), resulting in feelings of relief, satisfaction, and empowerment (Bensimon et al., 2008). Music-mediated excitation in the mesolimbic dopaminergic system may associate with reductions in negative cognition and mood improvements (Menon and Levitin, 2005; Salimpoor et al., 2011).

#### Hyperarousal

Hyperarousal includes high precaution, excessive startle responses, insomnia, irritability, aggression, lack of concentration, and lack of confidence (Shalev et al., 2017; Fenster et al., 2018). Listening to or playing music enables PTSD patients to create calmness, reduce tension, dismiss distressing reminders, and engages traumatized individuals to promote relaxation, perceive safety, and enjoy happiness (Orth, 2005; Macfarlane et al., 2019). Hyperarousal is reported to be associated with increased amygdala activity, while musical intervention is found to calm amygdalar actions that reduce hyperarousal (Hayes et al., 2012; Pitman et al., 2012).

## Discussion

### Music therapy on PTSD associated with VTA dopaminergic action

The auditory cortex is the primary center for musical information processing, which carries out musical perceptual analyses and extracts abstract information from acoustic features (such as pitch, timbre, intensity, and roughness) (Koelsch and Siebel, 2005; Boso et al., 2006). Interestingly, acoustic projections to several areas of the limbic system, including the VTA (Koelsch and Siebel, 2005; Menon and Levitin, 2005; Kraus and Canlon, 2012), are associated with emotional modulation. Accumulating evidence illustrates that the auditory cortex causes functional modifications to the VTA through complicated projections (Salimpoor and Zatorre, 2013; Belfi and Loui, 2020).

**Figure 2:**
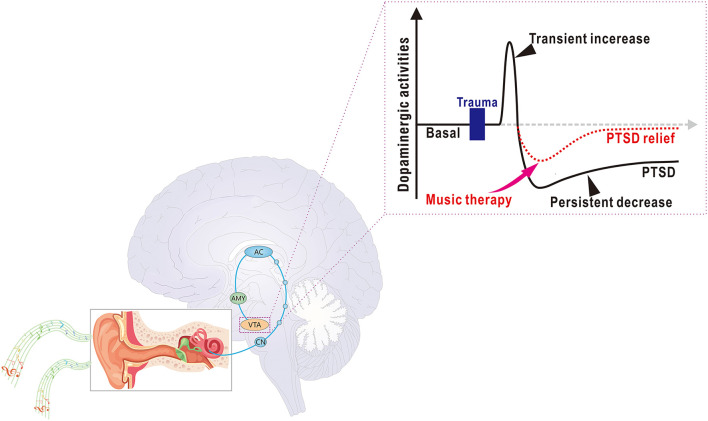
Schematic illustration of music therapy on post-traumatic stress disorder (PTSD) through dopaminergic action. A simplified neural pathway for acoustic processes from the cochlea to the auditory cortex. The auditory cortex sends projections to the amygdala and then to the ventral tegmental area (VTA) (Koelsch, 2014). Inset, the DA level is transiently elevated after trauma, followed by persistent hypodopaminergia (Corralfrias et al., 2013). Music activates the VTA function and stimulates the DA release, which may reduce PTSD-induced hypodopaminergia in the brain, which ameliorates PTSD symptoms. CN, cochlear nucleus; AC, auditory cortex; AMY, amygdala; VTA, ventral tegmental area.

The VTA has been shown to be activated due to music listening, and consequently, DA release is triggered (Menon and Levitin, 2005; Salimpoor et al., 2011). Several studies have revealed that PTSD was associated with reduced VTA dopaminergic activities and DA levels in the brain (Corralfrias et al., 2013). Interestingly, some medications that stabilize or enhance dopaminergic signaling in the brain can ameliorate PTSD symptoms in patients (Houlihan, 2011; McLaughlin et al., 2016; Zhou et al., 2021). A previous review summarizes that VTA dopaminergic activation rescues PTSD symptoms through several principal pathways, such as the VTA dopaminergic projections to the NAc, PFC, hippocampus, habenula, and amygdala; all of which are strongly involved in PTSD-related symptomatic processes (Zhou et al., 2021). Therefore, musical activation of VTA dopaminergic singling may also replicate actions of the pathways that theoretically compensate for PTSD-induced hypodopaminergia and conjecturally mitigate PTSD symptoms (Figure 2). This hypothesis is in line with previous theories: when an individual is confronted with traumatic events, the DA level is transiently increased, but followed by a persistent decrease (Corralfrias et al., 2013), and if the dopaminergic neurons are re-excited after the trauma, they may send safety signals to the fear circuits, which may prevent PTSD development (Lee et al., 2016). Nevertheless, the precise mechanism underlying music-mediated amelioration of PTSD symptoms through VTA dopaminergic action deserves further investigation.

Several other lines of evidence may also support the mitigation of PTSD symptoms *via* musical therapy by affecting mesolimbic dopaminergic actions. For example, PTSD is featured with anhedonia, which may associate with deficits in reward functioning (Enman et al., 2015), whereas music-stimulated VTA dopaminergic activation involves reward-value coding by organizing music into precise reinforcements (Menon and Levitin, 2005; Stegemöller, 2014; Zatorre, 2015). In addition, PTSD is also accompanied with social dissociation, while VTA dopaminergic activation promotes prosocial interactions (Hung et al., 2017; McHenry et al., 2017; Harvey, 2020; Wang et al., 2021), such as the enhancement of interpersonal trust, positive cooperation, and social connectedness among individuals (Chanda and Levitin, 2013; Harvey, 2018), which may improve symptoms in PTSD patients.

### Strengths and limitations of the present review

To our knowledge, the present review is the first to summarize the effects of music therapy on PTSD associated with VTA dopaminergic action; that is, we assessed whether music enhances VTA dopaminergic action, which compensates for PTSD-induced hypodopaminergia and consequently ameliorates PTSD symptoms. This review provides suggestions for clinicians and/or policymakers to develop standardized guidelines for the use of music therapy on PTSD. In addition, although this review substantiates a role for music therapy on PTSD symptoms associated with VTA dopaminergic action, the precise mechanism underlying the process remains largely enigmatic. It is our hope that the present review will inspire additional studies that evaluate the use of music therapy on PTSD.

Notably, the current review has several limitations. First, the paper was not prepared following a rigorous methodology of Preferred Reporting Items for Systematic Reviews and Meta-Analyses due to the sample size. We searched 19 original articles concerning music therapy on PTSD, six of which reported cases or without controls, and eight included small populations (only 5–16) of participants. Small sample sizes may result in substantial heterogeneity among participants and result in heterogeneity in the intervention effects (de Witte et al., 2022). Second, it is challenging to reach a standardized protocol for music therapy on PTSD due to the diverse methods employed across investigations, such as music intervention (receptive listening vs. active creation), music induction (prerecorded music *vs*. live music), music selection (pre-selected by the therapist vs. preferred by the patients), and music familiarity (familiar music vs. unfamiliar music) (Landis-Shack et al., 2017; Pant et al., 2022). Third, the unavoidable factor of publication bias could also affect the accuracy of the conclusions, as positive and significant outcomes are more frequently published relative to the non-significant or negative results (de Witte et al., 2019, 2022); i.e., the results reported in publications do not represent all findings.

## Conclusions

This review supports that music serves as an effective approach for treating PTSD that is associated with VTA dopaminergic activation. It should be noted that music therapy affects various aspects of physiological functions, such as various other brain circuits [e.g., mPFC-amygdala neurocircuit (Koelsch, 2014; Reybrouck et al., 2021)], neurotransmitters [e.g., oxytocin (Beck et al., 2018a; Harvey, 2020)], and immunological responsiveness [e.g., the reaction of the HPA axis (Reybrouck et al., 2018; Pant et al., 2022)], in addition to dopaminergic activities. Whether and how these different factors coordinate with VTA dopaminergic tone to treat PTSD symptoms warrants further investigation. Perhaps, music therapy could be combined with other methods to provide better therapeutic outcomes of PTSD intervention.

## Author contributions

MN and CZ conceived and designed the manuscript. MN, SW, and PZ searched and investigated the literatures. PZ and SW prepared the original draft. PZ and CZ wrote the paper. All authors read and approved the final manuscript.

## Funding

This work was supported by grants from the National Natural Science Foundation of China (81871062) and the Natural Science Foundation of Guangdong Province (2019A1515010331), and the key Project of Humanities and Social Sciences of Anhui Provincial Education Department (2017SK27).

## Conflict of interest

The authors declare that the research was conducted in the absence of any commercial or financial relationships that could be construed as a potential conflict of interest.

## Publisher's note

All claims expressed in this article are solely those of the authors and do not necessarily represent those of their affiliated organizations, or those of the publisher, the editors and the reviewers. Any product that may be evaluated in this article, or claim that may be made by its manufacturer, is not guaranteed or endorsed by the publisher.
